# Special Issue “Molecular Research on Platelet Activity in Health and Disease 2024”

**DOI:** 10.3390/ijms26083873

**Published:** 2025-04-19

**Authors:** Maria Valeria Catani, Isabella Savini, Valeria Gasperi

**Affiliations:** Department of Experimental Medicine, Tor Vergata University of Rome, 00133 Rome, Italy; savini@uniroma2.it

Platelets, once viewed primarily for their role in hemostasis, are now recognized to play a pivotal role in a broad spectrum of physiological and pathological processes, including thrombosis [1], inflammation [2], immunity [3], angiogenesis [4,5], and cancer metastasis [4,6]. The dynamic nature of these small, anucleate cells and their intricate molecular mechanisms have become a focal point of intense research. The present Special Issue, “Molecular Research on Platelet Activity in Health and Disease 2024”, compiles five key studies that significantly advance our understanding of platelet biology at the molecular level. These studies collectively deepen our knowledge of platelet function across diverse contexts, including cardiovascular disease, infectious syndromes, cancer progression and the beneficial effects of lifestyle changes. By thematically linking these findings, we highlight their broader implications for developing novel diagnostic and therapeutic strategies targeting platelet activity.

Two studies focus on the regulation of platelet activity in cardiovascular contexts. Hong and colleagues [7] explored the anti-platelet effects of daidzein, a soy isoflavone [8], demonstrating its ability to inhibit collagen-induced platelet aggregation through suppression of thromboxane A_2_ production and granule release. Daidzein modulates key signaling pathways like PI3K/Akt and MAPK while elevating cAMP levels, thus suggesting therapeutic potential for atherosclerosis and thrombosis. Ramirez and collaborators [9] introduced an innovative antibody targeting the extracellular loop of the serotonin 5-HT2A receptor, which selectively inhibits serotonin-enhanced platelet activation without impairing normal hemostasis. This approach, avoiding deleterious side effects of traditional antagonists [10,11,12], prolongs thrombus occlusion time, representing a promising strategy for thrombogenesis-related cardiovascular disorders.

The study by Makena and collaborators [13] investigated platelet-related biomarkers for monitoring health improvements following lifestyle changes, specifically smoking cessation. Within two weeks of abstinence, significant reductions in thromboxane metabolites (e.g., 2,3-d-TXB_2_) and leukotriene 4 (LTE_4_), alongside decreases in white blood cell and neutrophil counts, were observed. This highlights the rapid recovery potential of platelet-related biomarkers and their utility in monitoring physiological recovery after smoking cessation.

Freitag’s group identified a novel role for nucleosome assembly protein 1-like 1 (NAP1L1) in platelets and megakaryocytes [14]. They revealed that NAP1L1 dynamically interacts with mitochondrial enzymes, such as PDC-E2 (the E2 component of the mitochondrial pyruvate dehydrogenase (PDH) complex), to regulate cellular respiration and ATP synthesis. Elevated NAP1L1 levels correlate with increased PDH activity in septic patients, while overexpression disrupts thrombopoiesis by impairing proplatelet formation. This implies NAP1L1 is a key regulator of mitochondrial function and platelet production, offering insights into its role in infectious syndromes.

Finally, our group elucidated the intricate interplay between platelets and cancer cells, focusing on platelet-derived microvesicles enriched with miR-126-3p [15]. We identified AKT2 kinase as a direct target of miR-126-3p and demonstrated that this microRNA suppresses proliferation and invasion in breast cancer cells, particularly triple-negative subtypes, by modulating the PI3K/AKT signaling cascade. Notably, the association of high levels of miR-126 and low levels of AKT2 with favorable long-term prognosis in breast cancer patients underscores the potential of miR-126-3p as a prognostic biomarker, and suggests that targeted manipulation of platelet-derived microvesicle delivery could represent a promising avenue for enhancing therapeutic strategies against tumors.

In summary, this Special Issue highlights the multifaceted roles of platelets across various physiological and pathological processes. Each study provides a unique piece to the complex puzzle of platelet biology, offering novel insights into molecular mechanisms and potential therapeutic targets. From the cardiovascular benefits of daidzein [7] and the innovative antibody targeting the serotonin 5-HT2A receptor [9] to the diagnostic potential of platelet biomarkers in monitoring smoking cessation [13], the regulatory role of NAP1L1 in mitochondrial function and platelet production [14], and the anti-tumor effects of platelet-derived microvesicles [15], these findings collectively underscore the importance of continued research into the complicated roles of platelets. As we move forward, translating these molecular insights into clinical applications will be crucial for improving patient outcomes across diverse medical fields, paving the way for targeted therapies and personalized medicine approaches that harness the potent, yet potentially dangerous, capabilities of these small vital cells.
